# Protein Supplementation during Mid-Gestation Alters the Amino Acid Patterns, Hepatic Metabolism, and Maternal Skeletal Muscle Turnover of Pregnant Zebu Beef Cows

**DOI:** 10.3390/ani12243567

**Published:** 2022-12-16

**Authors:** Javier Andrés Moreno Meneses, Karolina Batista Nascimento, Matheus Castilho Galvão, German Darío Ramírez-Zamudio, Tathyane Ramalho Santos Gionbelli, Marcio Machado Ladeira, Marcio de Souza Duarte, Daniel Rume Casagrande, Mateus Pies Gionbelli

**Affiliations:** 1 Department of Animal Science, Universidade Federal de Lavras, Lavras 37200-900, MG, Brazil; 2 Department of Veterinary Medicine and Animal Science, Universidad de Ciencias Aplicadas y Ambientales, Cartagena 130001, Bolivar, Colombia; 3 Department of Animal Bioscience, University of Guelph, Guelph, ON N1G 2W1, Canada

**Keywords:** amino acids, gene expression, gluconeogenesis, maternal nutrition, skeletal muscle

## Abstract

**Simple Summary:**

Pregnant beef cows raised on extensive pasture systems in tropical regions commonly experience nutrient restriction during gestation. Herein, it was postulated that a strategic maternal protein supplementation (PS) might reduce maternal tissue mobilization intensity. Therefore, this study aimed to assess how protein supplementation (~40% of crude protein at the level of 3.5 g/kg BW/day, achieving 12% CP) for cows fed low-quality forage (corn silage + sugarcane bagasse, achieving 5.5% of crude protein) affects the maternal blood profile and the mRNA abundance of skeletal muscle turnover markers and glycogenic enzymes in the liver. This study also aimed to assess if these responses were related to the fetal sex. The PS resulted in a greater nutritional status, which in turn was demonstrated by the greater insulin, IGF1, and glucose concentrations. The effects of PS in mid-gestation persisted into late pregnancy. Protein supplementation also enhanced hepatic gluconeogenesis from AA and altered skeletal muscle turnover. In conclusion, protein supplementation during mid-gestation promotes a greater hepatic glucose production from AA, preserving the maternal tissue reserves and benefiting the longevity of the cows of the herd.

**Abstract:**

From 100 to 200 days of gestation, 52 cows carrying male (*n* = 30) or female (*n* = 22) fetuses were assigned to CON (basal diet—5.5% of CP, *n* = 26) or SUP (basal diet + protein supplement [40% CP, 3.5 g/kg BW]—12% of CP, *n* = 26) treatments. Glucose concentrations decreased at 200 (*p* ≤ 0.01; CON = 46.9 and SUP = 54.7 mg/dL) and 270 days (*p* ≤ 0.05; CON = 48.4 and SUP = 53.3 mg/dL) for CON compared to SUP. The same pattern occurred for insulin (*p* ≤ 0.01). At parturition, the NEFA concentration was greater (*p* = 0.01, 0.10 vs. 0.08 mmol/L) for CON than for SUP. Total AA increased in SUP (*p* ≤ 0.03) at mid- and late-gestation compared to CON. At 200 days, CON dams carrying females had less essential AA (*p* = 0.01) than cows carrying males. The SUP dams had greater expressions of protein synthesis markers, namely *eIf4E* and *GSK3β* (*p* ≤ 0.04), at day 200 and of *MuFR1* (protein degradation marker, *p* ≤ 0.04) at parturition. Supplemented cows had higher hepatic pyruvate carboxylase expressions (*p* = 0.02). Therefore, PS alleviates the restriction overload on maternal metabolism.

## 1. Introduction

Pastures are often the basal feed for beef cattle in subtropical and tropical regions [1]. However, these systems are subject to seasonal changes in forage supply and quality [2]. Concerning breeding herds, in more technical production systems, the breeding season coincides with the rainy period [3]. Consequently, mid-to-late-gestation overlaps with the dry season [4], exposing pregnant beef cows and their fetuses to lower nutrient availability [5].

Consequently, pregnant beef cows fed below their maintenance requirements need to conserve energy through metabolic adaptations of hepatic and skeletal muscle tissue [6]. This condition leads to a greater intensity of maternal peripheral mobilization, especially of lean mass [7]. This occurs because the use of some substrates from maternal energy reserves (such as long-chain fatty acids, non-esterified acids, and ketoacids) is limited by the placenta [8]. In this sense, amino acids from maternal skeletal muscle tissue may be partitioned to improve fetal access to amino acids and glucose [9]. The glucose required by gestational tissues is, in turn, supported by the increased hepatic uptake of endogenous substrates [10,11]. In such a scenario, supplementation programs (using rumen-degraded or undegraded protein, or non-protein nitrogen sources) may be used for nutritional correction proposes [6,12,13]. Nitrogen-based supplementation improves low-quality pasture digestibility, leading to positive associative effects on the dry matter intake of ruminants [14,15,16]. This condition reflects greater nutrient availability for maternal gain, improving the pregnant component’s weight (as a fetus, amniotic fluid, placenta, and uterus) and the calf’s birth weight [17,18]. Moreover, the mid-gestation represents an opportunity to perform strategic protein supplementation for breeding herds. In this period, the maternal requirements are more discreet than in the early- and late-gestation, when the energy and nutrient requirements are intense to attend the mammary gland’s demand for milk production, and when there is a greater fetal demand for energy and nutrients for anabolism, respectively [19].

Additionally, there is a sex bias related to fetal development in mammals [20], suggesting a maternal dominance investment [21]. In other words, dams may ‘sense’ their fetal sex through physiological signals involving maternal–fetal systems [17,22]. Thus, dams may put more or less maternal inputs according to their fetuses’ sex to favor the one that will provide a higher marginal return under such scenarios (i.e., favorable or unfavorable nutritional conditions) [17,21,23].

Hence, in this study, it was postulated that protein supplementation from days 100 to 200 of gestation for cows fed low-quality pastures will amortize the maternal tissue mobilization intensity and preserve maternal tissue reserves over the supplementation period. It was assumed that strategic protein supplementation would prepare the maternal skeletal muscle for intense tissue mobilization to sustain the high fetal demand for amino acids and glucose in the last trimester of gestation without causing significant maternal negative impacts. Lastly, it was predicted that such changes would occur dependent upon fetal sex. Therefore, this study aimed to evaluate how protein supplementation during mid-gestation would affect the blood concentration of maternal amino acids and metabolites and the expression of transcription factors related to the turnover of skeletal muscle and liver glycogenic enzymes.

## 2. Materials and Methods

### 2.1. Experimental Design and Management

The Brazilian Ethics Committee on Animal Use (CEUA/UFLA—a process nº. 015/17) approved all experimental procedures of this trial. The study covered two years. Both repetitions had the same experimental procedures. Details of the experimental design were previously described by Nascimento et al. [18] and Meneses [24]. Briefly, 52 multiparous Tabapuã beef cows (*Bos taurus indicus*, yr 1: *n* = 25; yr 2: *n* = 27) were inseminated. At 60 days of gestation, fetal sexing was performed by transrectal ultrasonography, identifying 22 female and 30 male fetuses. From 0 to 100 days of gestation, all cows were managed as a single group on improved pasture (*Brachiaria brizantha* cv. Marandu) in a continuous stocking method with a variable stocking rate. All cows received an ad libitum mineral mixture during this period of early gestation. From ~100 days of gestation (mid-gestation onset), cows were housed in a tie-stall system equipped with individual pens (20 m^2^, roof covered (6 m), concrete floor), fitted with individual feeders and water bins. Cows remained housed in these individual pens during all mid- and late-gestation (i.e., from 100 days until calving).

At 100 days of gestation, the cows were fed the experimental treatments (Table 1) as follows: (1) control (CON)—basal control diet (75% of corn silage + 25% of sugarcane bagasse, plus mineral mixture, achieving 5.5% of CP, *n* = 26); or (2) protein supplementation (SUP)—basal diet plus a protein supplementation (40% CP; 3.5 g/kg of BW per day, achieving 12% of CP, *n* = 26).

The protein supplement was composed of 50% of soybean meal and 50% of commercial protein supplement (Probeef Sprint, Cargill Nutrição Animal, Itapira, SP, Brazil). During mid-gestation, cows were individually fed the basal diet ad libitum twice daily (0700 and 1300 h). The supplement offer (mineral or CP supplement) was delivered in the morning (0700 h). The basal diet offer was individually adjusted daily based on the refusals from the previous day feeding in order to keep ad libitum (around 5% of refusals) intake of roughage. The supplement supply adjustments were made based on the cow’s body weight. Thus, the pregnant beef cows were periodically weighed (once a month, after 16 h of fasting) to obtain such adjustments in the supplement supply. An average of 1.6 ± 0.8 kg of protein supplement (on a DM basis) was consumed daily per cow during the mid-gestation.

From 200 days of gestation until the parturition, all cows were ad libitum fed with corn silage and mineral supplement. Therefore, cows were fed differently only during mid-gestation. During late-gestation, the pregnant beef cows were also fed twice daily (0700 and 1300 h) and had free access to clean water. The amount of diet provided was adjusted for DM content weekly, based on the DM content of the roughage source. 

### 2.2. Measurements

#### 2.2.1. Blood Parameters

Blood samples (10 mL) were collected at 200 and 270 days of gestation. Before the morning feeding (0700 h), samples were taken from the jugular vein using vacutainer tubes with EDTA to prevent blood coagulation. After collection, samples were placed on ice, centrifuged at 2700 G for 20 min, and stored at −20 °C.

Blood glucose analyses were performed by a colorimetric method. Concentrations of glucose were quantified using kit No 133 (Glucose Liquiform, Labtest^®^, Lagoa Santa, Brazil; sensitivity: 1.77 mg/dL; precision: intra-assay: CV < 1.2%; inter-assay: CV < 2.2%), according to the manufacturer’s recommendations. Plasma urea-N concentrations were determined using a commercial (Bioclin, Química Básica Ltd.a, Belo Horizonte, MG, Brazil) kit (No K056; sensitivity: 0.175 mg/dL; precision: intra-assay: CV < 3.5%; inter-assay: CV < 1.9%) by the enzymatic method. Briefly, urea was hydrolyzed by urease, promoting the release of ammonia and CO_2_. Ammonia, in turn, reacted with 2-oxoglutarate and NADH (via catalysis by glutamate dehydrogenase) with the oxidation of NADH and NAD^+^, with the absorbance decrease being proportional to the urea concentration [25]. Serum concentrations of D-3-hydroxybutyrate (BHBA) were determined by the enzymatic kinetic method using a commercial (Randox Laboratories Ltd. Antrim, United Kingdom) kit (No RX Monza RB 1007, sensitivity: 0.100 mmol/L; precision: intra-assay: CV < 4%; inter-assay: CV < 5.2%), according to the manufacturer’s guidelines. This method considers the D-3-hydroxybutyrate oxidation into acetoacetate through the 3-hydroxybutyrate dehydrogenase action, which reduces the NAD+ cofactor to NADH, with the absorbance values being directly related to BHBA concentration [26]. Concentrations of non-sterilized fatty acids (NEFA), insulin, and insulin-like growth factor were determined using the ELISA test. The NEFA concentration was determined using a Bovine non-ester fatty acid (Bioassay Technology Laboratory, Yangpu Dis., Shanghai, China) kit (No E0419Bo, sensitivity: 1.02 µmol/L; precision: intra-assay: CV < 8%; inter-assay: CV < 10%). Insulin concentrations were determined using a commercial (Monobind Inc., Lake Forest, CA, USA) test (No 2425-300; sensitivity: 0.182 µIU/mL; precision: intra-assay: CV < 8.3%; inter-assay: CV < 11.3%) following the company’s recommendations. The enzyme immunoassay for IGF-1 concentration was determined using a Bovine IGF-1 (Bioassay Technology Laboratory, Yangpu Dis., Shanghai, China) kit (No E0016Bo; sensitivity: 0.53 ng/mL; precision: intra-assay: CV < 8%; inter-assay: CV < 10%).

The high-performance-liquid chromatography method (Model LC-MSMS TQD, Waters, Milford, MA, USA) was used to determine the blood AA concentrations. An analysis was performed in a commercial laboratory (Viçosa LAB laboratory, Viçosa, MG, Brazil). Amino acids were individually evaluated, being classified as essential (Arginine, Phenylalanine, Histidine, Isoleucine, Methionine, Leucine, Tryptophan) or non-essential AAs (Aspartate, Glutamate, Asparagine, Serine, Glutamine, Tyrosine, Alanine), and also grouped according to their functions (branched-chain AA, glucogenic, and ketogenic). 

#### 2.2.2. Skeletal Muscle and Liver Tissue Sampling

Skeletal muscle and liver tissue samples were biopsied from each animal at 200 and 270 days of gestation. Six mL of lidocaine (2%) was applied per animal (4 and 2 mL in the muscle and liver sites, respectively). The muscle tissue samples (~1 g) were obtained from the Longissimus Dorsi muscle between the 12th and 13th ribs of the right side, while the liver samples were obtained at the 11th intercostal space of the right rib cage. The biopsied regions were previously disinfected with 70% alcohol before the biopsy. The muscle tissue samples were obtained through percutaneous biopsies with Bergström-type needles (Stille, Ekbacken, Thorshälla, Sweden). The liver samples were collected using a Tru-Cut needle (ProMedical Equipamentos Médicos Ltd, Juiz de Fora, Minas Gerais, Brazil), as described by Engle et al. [27]. All muscle and liver samples were immediately placed in 2 mL cryovial, snap-frozen in liquid nitrogen, and stored at −80 °C until analysis.

#### 2.2.3. Gene Expressions Analysis

Genes that encode the following proteins, namely, ribosomal protein S6 kinase (*p70S6k*), glycogen synthase kinase 3β (*GSK3B*), eukaryotic translation initiation factor 4E (*eIf4E*), muscle ring finger 1 (*MuRF1*), and muscle atrophy F-box protein (*Atrogin-1*), were elicited to be evaluated in this study (Table 2). These molecular markers play a central role in the skeletal muscle protein turnover, being indicative of maternal muscle protein breakdown and synthesis. Genes that encode some key enzymes of the gluconeogenesis pathway in the liver tissue, namely, propionyl-CoA carboxylase (*PCCA*), pyruvate carboxylase (*PC*), and phosphoenolpyruvate carboxykinase 1 (*PCK1*) (Table 2) were evaluated. The enzyme PCCA is involved in the conversion of propionate to glucose [28]. The PC enzyme catalyzes the carboxylation of pyruvate to form oxaloacetate [29]. Lastly, PCK1 is, in turn, responsible for catalyzing the reaction of oxaloacetate and guanosine triphosphate (GTP) into phosphoenolpyruvate, guanosine 5′-diphosphate (GDP), and CO_2_ [30]. Genes that encode IGF-1 and GH receptors in the liver were also evaluated to understand the impacts of maternal protein supplementation on the anabolic process and energetic metabolism.

All samples obtained in the biopsies (*n* = 52 muscle samples and *n* = 52 liver samples) were analyzed by the PRC-RT technique. Total RNA extraction from liver and muscle samples was performed using QIAzol (QIAGEN, Valencia, CA, USA). Samples were treated with DNA-free DNase (Ambion, Austin, TX, USA) according to the manufacturer’s instructions. To evaluate RNA integrity, the 28S and 18S rRNA bands were analyzed.

The total RNA was electrophoresed in a 1.0% (*m*/*v*) agarose gel, stained with GelRed nucleic acid gel stain (Biotium, Hayward, CA, USA), and visualized with a UVItec FireReader XS D-77Ls-20M (UVItec, Cambridge, UK). The cDNA synthesis was performed using the High-Capacity cDNA Reverse Transcription Kit (Applied Biosystems, Foster City, CA, USA) according to the manufacturer’s instructions, and samples were stored at −20 °C. A reverse-transcription quantitative PCR (RT-qPCR) was performed on the Eppendorf Realplex System (Eppendorf, Hamburg, Germany) using the SYBR Green detection system (Applied Biosystems, Foster City, CA, USA).

The relative mRNA expression was determined using the threshold cycle (CT) method, using the primers of each gene. The *β-actin* and *GAPDH* (*D-glyceraldehyde-3-phosphate dehydrogenase*) were used as reference genes for an expression analysis for liver samples, while *β-actin* and *CASC3* (*Cancer susceptibility candidate 3*) were used as reference genes for skeletal muscle samples [6,31] samples.

Primers for target gene amplification and endogenous amplification were designed using the sequences available in the GenBank database (Primer Quest Tool of Oligo Perfect™ Design software, Table 2). The PCR primers were commercially synthesized (Life Technologies, São Paulo, BR) and reconstituted to a final concentration of 10 μmol/L.

### 2.3. Statistical Analyses

All analyses were performed using SAS 9.4 (Statistical Analysis System Institute, Inc., Cary, NC, USA). The maternal nutrition plan (CON or SUP), calf sex (male or female), and interactions among these factors were considered fixed effects. The year of study (y1 or y2) was considered a random effect in the model, as follows:

*Y_ijk_* = *μ* + *D_i_* + *S_j_* + *F_k_* + (*DS*)*_ij_* + (*BW*)*_ijk_* + *e_ijk_*

where *Y_ijk_* is the observed measurement; *µ* is the overall mean; *D_i_* is the fixed effect of the *i*th level of maternal dietary treatment (2 levels); *S_j_* is the fixed effect of the *j*th level of calf sex (2 levels); *F_k_* is the random effect or the *F*th level of the year (2 years); *DS_ij_* is the interaction between *D* and *S*; *BW_ijk_* is the covariate of initial empty body weight, initial BCS, gestation time, and parity of the dam (when pertinent); and *e_ijk_* is the random error associated with *Y_ijk_*, with *e_ijk_ ~ N* (0,*σ_e_*^2^).

To determine the normality achievement, the mRNA expression levels were converted using the natural logarithm of the expression values þ1. A Shapiro–Wilk’s test was used to assess the normality distribution of the data set. Least-squares means were separated using Fisher’s least significant difference test. Results were deemed significant when *p*-value ≤ 0.05, and trends when 0.05 < *p*-value ≤ 0.10.

## 3. Results

### 3.1. Maternal Performance and Voluntary Feed Intake

At the beginning of the experimental period (day 100 of gestation), all cows had similar body weights (*p* = 0.67; CON = 486 ± 22.3 kg and SUP = 496 ± 23.7 kg (means ± SEM)). At the end of the supplementation period (day 200 of gestation), CON cows were 78 kg lighter (*p* < 0.01) compared to SUP cows. The average BW was 470 ± 4.9 kg for CON and 548 ± 5.5 for SUP. At pre-parturition (day 270 of gestation), SUP cows remained heavier than CON (*p* < 0.01; CON = 490 ± 5.6 kg and SUP = 557 ± 6.2 kg).

**Figure 1:**
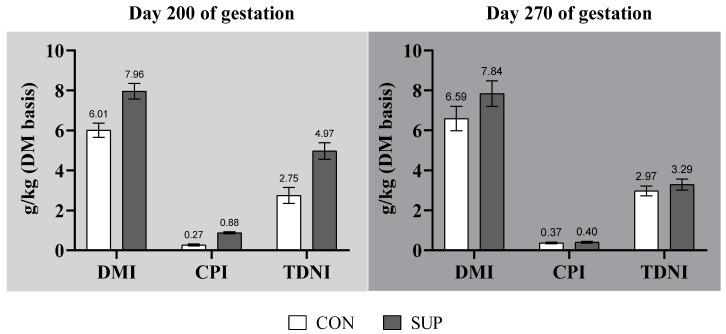
Effects of maternal feeding regimen on the dry matter intake (DMI), crude protein intake (CPI), and total digestible nutrients intake (TDNI) at 200 and 270 days of gestation.

The total DM, CP, and TDN intakes during mid-gestation were greater *(p* < 0.01) for SUP cows (Figure 1). At late-gestation (day 200 to parturition), the total DMI was ~19% greater for SUP cows (*p* = 0.01). Nevertheless, no effects of the maternal feeding regimen were verified on the CP or TDN intakes (*p* ≥ 0.34). No MN × CS interactions were verified for these outcomes (*p* ≥ 0.10) at mid- or late-gestation.

Based on the pregnant beef cow’s voluntary feed intake and diet-analyzed nutrient profile at mid-gestation (day 100 to 200 of gestation), CON cows ingested, respectively, 30% and 50% of their protein and energy requirements, according to the Nutrient Requirements of Zebu and Crossbred cattle [19]. In contrast, during the same period, SUP cows ingested 98% and 92% of their protein and energy requirements, respectively. In the late-gestation (day 200 to pre-parturition), all cows (CON and SUP) were estimated to ingest 40% and 35% of their protein and energy needs [19], respectively.

### 3.2. Blood Parameters

There was no interaction between maternal nutrition and calf sex for the plasma concentration of hormones and metabolites (*p* ≥ 0.07; Table 3). Nevertheless, at day 200 of gestation, there was a tendency toward a greater additional plasma IGF-1 concentration for SUP cows compared to the CON group (*p* = 0.06). Concentrations of insulin, glucose, and IGF-1 in the plasma of SUP cows were higher (*p* ≤ 0.01) during 200 and 270 days of gestation compared with CON cows (Table 3). Concentrations of non-esterified fatty acids and urea on day 200 were similar between dietary treatments (*p* ≥ 0.10), but during the late-gestation, CON cows presented higher (*p* ≤ 0.01) plasma concentrations of NEFA (0.110 mmol/L vs. 0.078 mmol/L ± 8.07) and urea (35.44 mg/dL vs. 30.64 mg/dL ± 3.15), respectively. Concentrations of BHBA tended (*p* = 0.08) to be greater in SUP than in CON cows at mid-gestation.

**Figure 2:**
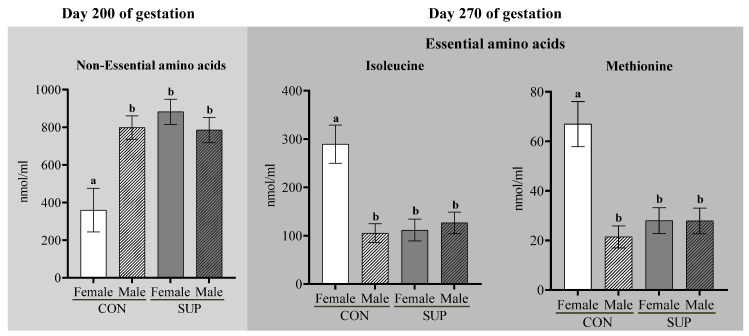
Interaction between dietary treatments and calf sex on the cows’ blood concentrations of non-essential AAs (200 d) and of isoleucine and methionine concentrations (270 d). Means followed by a different superscript are different (*p* < 0.05), by Tukey’s test. Bars represent means ± SEM. ^a,b^ are significant differences between the groups.

At 200 days of gestation, there was a significant MN × CS interaction for concentrations of non-essential AAs (*p* ≤ 0.01), with ~30% lower concentration observed in CON dams carrying female fetuses compared to the other groups (Table 4 and Figure 2).

At day 270 of gestation, there was a significant MN × CS interaction for branched-chain AAs, with ~60% higher concentrations in CON dams carrying female fetuses (*p* = 0.06; Table 4). At pre-parturition, SUP cows had ~50% and 27% other concentrations of total circulating Aas and glycogenic and ketogenic Aas than CON, respectively (*p* ≤ 0.01). The essential and non-essential Aas tended to be ~16% and ~31% higher in SUP cows (*p* ≤ 0.09) in the same period. Lower concentrations of ketogenic and branched-chain Aas were also observed in dams carrying male fetuses (*p* ≤ 0.05).

On day 200 of pregnancy, concentrations of phenylalanine tended (*p* = 0.08) to be higher, and those of serine and glutamine tended (*p* ≤ 0.10) to be lower in CON dams carrying female fetuses than in other groups. Concentrations of tryptophan, tyrosine, and alanine were ~54%, ~38%, and ~23% higher (*p* ≤ 0.03) in SUP than CON cows. On day 270, essential AAs such as phenylalanine, leucine, and tryptophan were ~32%, ~28%, and ~45% more abundant in SUP cows, respectively (*p* ≤ 0.03). Of the non-essential AAs, concentrations of tyrosine and alanine were ~48% and ~36% higher (*p *≤ 0.02) on day 200 in SUP than in CON cows. Additionally, regardless of the nutritional plane during mid-gestation, dams carrying male fetuses had a ~20% higher plasma tyrosine concentration on day 270 (*p* = 0.02; Table 5). There was also a MN × CS interaction on day 270 (*p* ≤ 0.05) for methionine and Isoleucine, such that concentrations were ~50% higher in CON cows carrying female fetuses than in other groups (Figure 2). 

### 3.3. Gene Expression in Skeletal Muscle and Liver Tissues

**Figure 3:**
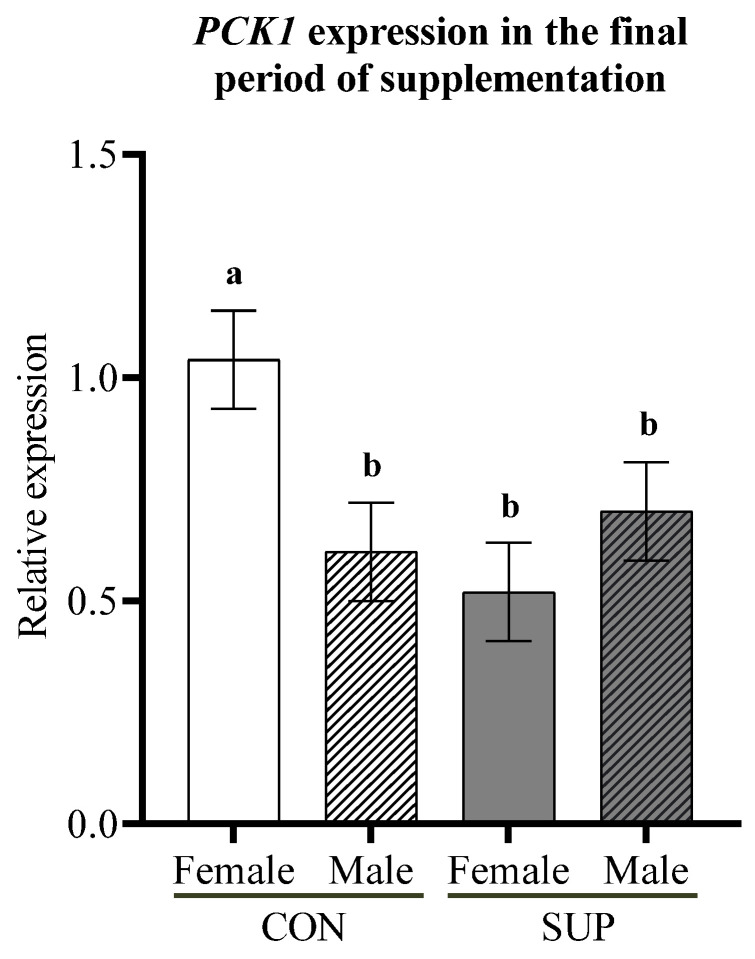
Interaction of maternal nutrition and fetal sex on the relative expression of PCK1 in liver from dams during the final period of supplementation. ^a,b^ are significant differences between the groups.

There was a trend toward higher mRNA abundance for *PCK1* in CON dams carrying female fetuses (*p* = 0.09) at pre-parturition (Figure 3).

**Figure 4:**
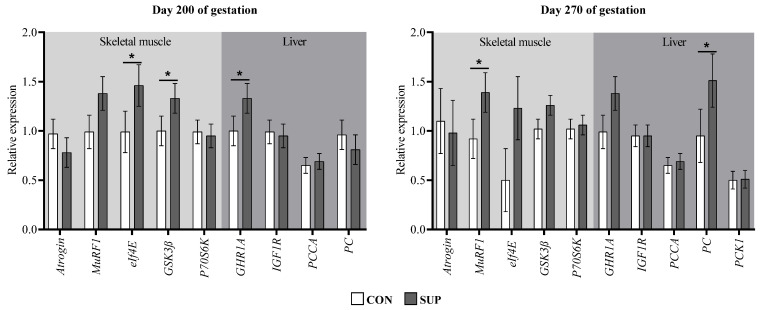
Gene expression in *longissimus thoracis* muscle and liver of cows with or without protein supplementation during mid-gestation. *Atrogin* = muscle atrophy F-box protein; *MuRF1* = muscle ring finger 1; *eIf4E* = eukaryotic translation initiation factor 4 E; *GSK3β* = glycogen synthase kinase 3β; *p70S6k* = ribosomal protein S6 kinase; *PCCA* = propionyl-CoA carboxylase; *GHR1A* = growth hormone receptor 1A; *IGF1R* = insulin-like growth factor 1 receptor; *PC* = pyruvate carboxylase; *PCK1* = phosphoenolpyruvate carboxykinase 1. Values with asterisks are significantly different (*p* ≤ 0.05) and π represents a tendency to be different (0.05 > *p* ≤ 0.10).

**Figure 5:**
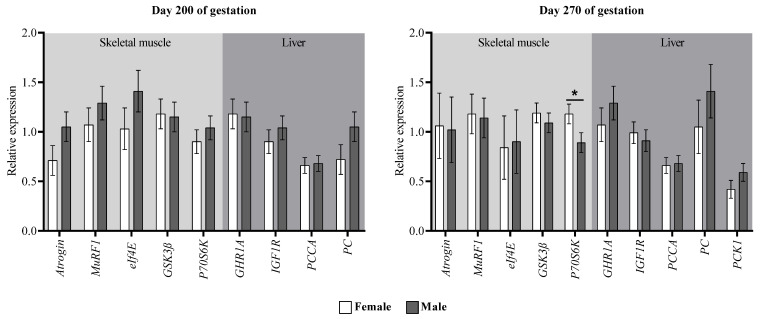
Gene expression in the *longissimus thoracis* muscle and liver of cows carrying female and male fetuses. *Atrogin* = muscle atrophy F-box protein; *MuRF1* = muscle ring finger 1; *eIf4E* = eukaryotic translation initiation factor 4 E; *GSK3β* = glycogen synthase kinase 3β; *p70S6k* = ribosomal protein S6 kinase; *PCCA* = propionyl-CoA carboxylase; *GHR1A* = growth hormone receptor 1A; *IGF1R* = insulin-like growth factor 1 receptor; *PC* = pyruvate carboxylase; *PCK1* = phosphoenolpyruvate carboxykinase 1. Values with asterisks are significantly different (*p* ≤ 0.05) and π represents a tendency to be different (0.05 > *p* ≤ 0.10).

At the end of the supplementation period, skeletal muscle from SUP cows showed higher *eIf4E* and *GSK3β* expressions compared to the CON group (*p* ≤ 0.04; Figure 4). It tended to remain higher expressed during the pre-parturition period (*p* ≤ 0.10). Additionally, SUP cows showed a trend of greater *MuRF1* expressions at day 200 of gestation (*p* = 0.09) and a greater abundance of mRNA of this same gene in the pre-parturition period (*p* = 0.04) compared to CON dams. In addition, at the end of gestation, regardless of the maternal nutritional plane, dams carrying male fetuses had a lesser abundance of *P70S6K* mRNA (*p* = 0.05; Figure 5). As for the genes in liver tissue, there was only a greater abundance of *PC* mRNA in cows of the SUP group during pre-parturition (*p* = 0.02).

## 4. Discussion

The effectiveness of treatment application was demonstrated through the CON and SUP beef cows’ BW differences. The variation sources related to maternal nutritional treatment were only related to the protein supply, being, in the CON and SUP diets, isoenergetic. As the CON and SUP diets were isoenergetic, differences in maternal nutrition were solely due to the protein concentration of the diet. Thus, maternal DMI was greater in SUP than CON cows, because DMI was suppressed in the cows with inadequate dietary protein. Control cows (fed low-quality forage) presented a lower dry matter intake because the nitrogen scarcity in the rumen environment limited microbial protein synthesis, promoting a ‘filling effect’ and compromising the digesta passage rate. Therefore, the supplementation program utilized was able to promote a positive associative effect on the forage intake. Such a response is well-known in the field of animal nutrition [32,33].

The development and function of fetal tissues depend on the partition of nutrients due to maternal nutritional status [34]. The adequate metabolic state of SUP cows was demonstrated by higher concentrations of glucose, IGF-1, insulin, and low plasma BHBA and NEFA [35], consistent with the blood parameters found in SUP cows in the present study. In a normal state, fetal arterial glucose concentration and consumption in the uterus and placenta are defined by the maternal concentration of this metabolite [36,37]. Although in the mid-gestation of CON dams carrying female fetuses there was a trend of the upregulation of *PCK1* that encodes the enzyme responsible for the initial stage of hepatic gluconeogenesis [30], the plasma glucose concentration was lower in CON cows. A reduced glucose concentration in these cows likely leads to catabolism or transamination for fetal gluconeogenesis, which explains the lower concentration of glycogenic amino acids in CON cows [37].

The supply of AAs to the fetus is determined by concentrations in maternal blood, and the uptake and metabolism of the AAs in the placenta. Fetal sex affects glucose uptake in the placenta, such that glucose transport is greater for male than female fetuses. Thus, the lower concentrations of SER in CON than SUP cows may reflect a high uptake of this AA for transamination as a compensatory mechanism in fetal gluconeogenesis [38,39,40,41].

Glutamine is an amino acid involved in multiple metabolic and physiological functions, mainly as a substrate for fetal muscle development, and also in the formation of glutamate as a fuel source for the placenta [42]. Consistently, under intrauterine growth restriction conditions during late-gestation in pigs, fetal glutamine concentration was reduced [43]. Additionally, the placental conversion of glutamate to α-ketoglutarate is limited, and a transformation of glutamine to glutamate in the fetal liver is preserved to maintain the supply of fuel to the placenta [42]. In a trial with pregnant sheep, it was shown that of the total glutamine passing through the fetal liver, ~45% was returned to the placental circulation in the form of glutamate [44]. Therefore, it is presumable that in a nutritional restriction, an increased flow of maternal glutamine into the fetal circulation is used to meet the glutamate requirements of the placenta. Consequently, lower glutamine concentrations in CON cows with a female fetus in the present study are likely due to a greater availability of this amino acid in the fetal circulation, and subsequent transformation into glutamate.

On the other hand, methionine is an amino acid that participates as a substrate for polyamine synthesis, which regulates placental angiogenesis [45], and is additionally a donor of methyl groups in one-carbon metabolism [46]. Methionine is required for S-adenosylmethionine production, which in turn is the substrate for methyltransferases to induce histone and DNA methylation, altering gene expression [47]. Higher plasma methionine concentration in CON cows with female fetuses in the present study may indicate a lower transport of this amino acid to the fetal-placental circulation. This indicates that dams carrying female fetuses that suffer nutritional restriction during pregnancy may have metabolic adaptations to provide a suitable maternal environment for fetal development. This response demonstrates that the effects of fetal programming on post-natal performance are dependent upon fetal sex, and that the impact of maternal nutrition is more expressive in male fetuses [23]. Therefore, the present findings support the suggestion of the Trivers and Willard theory [24] that the maternal environment favors female fetuses due to their greater reproductive relevance in the preservation of the species.

Alanine is one of the main amino acids of hepatic gluconeogenesis in cattle [48], and in fetal muscle, it is also used as a proteinogenic amino acid [49]. Under normal nutritional conditions, the flux of maternal alanine into fetal circulation is negligible due to a high turnover of this amino acid in the placental tissue [38]. However, in nutritionally restricted ovine fetuses, hepatic gluconeogenesis to meet energy expenditure is carried out through the glucose–alanine cycle [50,51]. In this condition, an increase in alanine utilization can lead to an excess in the placental nitrogen excretion, which can be solved through three pathways: an increase in ammonia excretion, the transformation of glutamate into glutamine by the amidation mechanism, or a reduction of the deamination of branched-chain amino acids [38]. It is likely that of these placental nitrogen clearance pathways, the last was more active during pre-parturition in non-supplemented dams carrying female fetuses in the present study, supported by the tendency to increase maternal urea and branched-chain amino acid concentrations.

The tricarboxylic acid cycle (TCA) in mammals is a mechanism for energy production from the oxidation of acetyl-CoA, and that also provides intermediate metabolites for pathways such as gluconeogenesis and fatty acid production [52]. In ruminants, propionate is the main carbon donor for glucose production, entering the TCA cycle in the form of succinate [53]. However, when the glucose expenditure exceeds the ruminal supply of propionate, lactate and amino acids, mainly alanine, enter the TCA cycle in the form of oxaloacetate through the activity of the enzyme pyruvate carboxylase [53]. Additionally, in models of feed restriction during late-gestation and early-lactation in dairy cows, an increase in pyruvate carboxylase expression is a mechanism to increase glucose availability from alanine and lactate [54]. In contrast, the present study showed that non-supplemented cows at mid-gestation had a lesser abundance of mRNA encoding the enzyme pyruvate carboxylase during pre-parturition. This can potentially be attributed to an indirect response of the lower alanine concentration observed in cows at 200 and 270 days of gestation in the present study. The amino acid alanine is an analog of pyruvate [55]. Pyruvate is the substrate for oxidation by pyruvate carboxylase for maintaining oxaloacetate in the TCA cycle [52]. A lesser alanine abundance is likely a signal to decrease pyruvate carboxylase synthesis. Furthermore, the patterns of BHBA and NEFA concentrations on days 200 and 270 in CON cows imply that there was a lack of anaplerotic supply of oxaloacetate, which reduced the oxidative capacity of acetyl-CoA [52], and the lower alanine concentration was likely to be a consequence.

Another fact contributing to the formation of ketone bodies is related to an increase in the use of amino acids that use the gluconeogenic/ketogenic pathway, such as tryptophan, tyrosine, and phenylalanine, or ketogenic amino acids such as leucine [56]. Studies in calorie-restricted rats have revealed that the transamination of tryptophan, tyrosine, and phenylalanine ultimately leads to an accumulation of acetyl-CoA [56,57,58]. In the present study, at the end of the supplementation period and in the pre-parturition period, CON dams showed a reduction in these aforementioned amino acids. In this sense, these amino acids were probably being used as another source for glucose formation in addition to alanine utilization. In contrast, Lopes et al. [6] found no significant effects on tryptophan, tyrosine, and phenylalanine concentrations between supplemented and non-supplemented (crude protein) beef cows during the last third of gestation. On the other hand, excessive gluconeogenesis is reported to deplete the oxaloacetate pool and consequently reduce the ability to fully oxidize acetyl-CoA in liver cell mitochondria [52,58,59]. Therefore, the circulating BHBA on day 270 in SUP cows probably reflects a greater production of glucose to meet fetal demands.

The GH/IGF1 axis is involved in a wide range of functions, mainly in cell growth and in the control of gluconeogenesis, which is mediated by GH receptors (GHR) [60]. In cows during the peri-partum, a reduction in the IGF1 release as a consequence of hepatic downregulation of *GHR1A* is due to a decrease in insulin [61]. The less abundance of *GHR1A* mRNA in the liver from CON dams in both evaluation periods, and a trend of lower plasma IGF-1 concentration on day 200 of gestation, are related to reduced insulin concentration. As an indicator of skeletal muscle turnover in the cows of the present study, the molecular markers *EIF4E* and GSK3β for synthesis, and *Atrogin* and *MuRF1* related to protein degradation were analyzed. At the end of the supplementation period, SUP cows had higher *EIF4E* and *GSK3β* expression and presented a trend toward a greater abundance of *MuRF1* mRNA. In the pre-partum period, these same dams showed a higher expression of *MuRF1* and a tendency to increase the abundance of mRNA for *EIF4E* and *GSK3β*. Consistently, in a complementary study with these same animals, a greater ribeye area was observed in SUP cows only during the final period of supplementation [24]. This shows that protein supplementation during mid-gestation prepares the dam with greater body reserves to be used in the period of the greatest nutritional demand of the fetus at the end of gestation. Research with rodents has shown that dams during early pregnancy have an anabolic phase, accumulating muscle protein reserves which are catabolized in late pregnancy to support the exponential growth of the fetus [62,63].

## 5. Conclusions

In conclusion, the protein supplementation for beef cows fed low-quality forage improves the AA and glucose availability for fetal development, preserving the maternal tissue reserves at mid-gestation. The supplementation program used in the current study was demonstrated to promote a beneficial effect on the maternal metabolism into late pregnancy, which in turn benefits the fetal development. Finally, there are effects upon maternal metabolism that are determined by the sex of the fetus and are associated with dietary perturbations over pregnancy, which indicate a greater maternal investment for female fetuses’ exposure to a low-protein maternal diet compared to males.

## Figures and Tables

**Table 1:** Nutrient composition of diets over gestational period.

Item	Mid-Gestation (Day 100 to 200)		Late-Gestation (Day 200 to Parturition)
	CON ^1^	SUP ^2^	
Ingredients			
Corn silage, % of DM	75	75	100
Sugarcane bagasse, % of DM	25	25	-
Supplement, kg of DM per day	-	1.6	-
Chemical composition			
Dry matter, g/kg of DM	418	492	330
Organic matter, g/kg of DM	951	960	941
Crude protein, g/kg of DM	53.3	125	72.2
Ash and protein-free neutral detergent fiber, g/kg of DM	631	51.6	549
Ether extract, g/kg of DM	24.1	29.4	29.2
Total digestible nutrients, g/kg of DM	614	647	677
Metabolizable energy, Mcal/kg	2.27	2.39	2.45

^1^ Control: corn silage + sugarcane bagasse. ^2^ Supplement: corn silage + sugarcane bagasse + protein supplement (50% of Probeef Proteinado Sprint^®^ + 50% soybean meal). Probeef Proteinado Sprint^®^ (Cargill Nutrição Animal, Itapira, SP, Brazil)—assurance concentrations per kilogram of product: 70 g Ca (max); 50 g Ca (min); 15 mg Co (min); 255 mg Cu (min); 15 g S (min); 2000 mg F (max); 20 g P (min); 15 mg I (min); 510 mg Mn (min); 340 NPN protein eq. (max); 450 g CP (min); 4 mg Se (min); 95 g Na (min); 850 mg Zn (min); 50 mg Flavomycin.

**Table 2:** Sequence, function, and NCBI^1^ access number of the primers used for quantitative PCR.

Gene	Gene Abbreviation	Function	Access Number	Primer
Ribosomal protein S6 kinase	*p70S6k*	Protein Synthesis	AY396564.1	F TTGAACCAAAAATCCGATCC
				R AGCACCTCTTCCCCAGAAA
Glycogen synthase kinase 3β	*GSK3B*	Protein Synthesis	NM_001101310.1	F GCCCAGAACCACCTCCTTT
				R TGCTGCCATCTTTGTCTCTG
Eukaryotic translation initiation factor 4 E	*eIf4E*	Protein Synthesis	NM_174310	F AAACCACCCCTACTCCGAAT
				R TGCCCATCTGTTCTGTAAAGG
Muscle ring finger 1	*MuRF1*	Protein Degradation	NM_001046155.1	F GGGACAGATGAGGAAGAGGA
				R CCTCATCATCGCCTTACTGG
Muscle atrophy F-box protein	*Atrogin-1*	Protein Degradation	NM_001046155.1	F CCTTGAAGACCAGCAAAACA
				R AGACTTGCCGACTCTTTGGA
Growth hormone receptor 1A	*GHR1A*	Anabolism	AY748827	F TCCAGCCTCTGTTTCAGGAG
				R GCTGCCAGAGATCCATACCT
Insulin-like growth factor 1 receptor	*IGF1R*	Anabolism/Energy Metabolism	NM_001077828	F ATGTACTGCGCGCCTCTC
				R CCCTCTACTTGTGTTCTTCAAATG
Propionyl-CoA carboxylase	*PCCA*	Gluconeogenesis	NM_001083509.1	F TTTGGTTTGCCGTCTGTTGG
				R TTGAATGCCGCTGTCAACTC
Pyruvate carboxylase	*PC*	Gluconeogenesis	NM_177946	F GAGGTGGTCCGCAAGATG
				R TCGTGCAGGGAAGTGATG
Phosphoenolpyruvate carboxykinase 1	*PCK1*	Gluconeogenesis	NM_174737.2	F GGGCTGATCGAAACCCTTAAT
				R TTTCCTGGAGCCTGCTATTTC
Β-actin	*Β-actin*	Endogenous Control	BC142413.1	F GTCCACCTTCCAGCAGATGT
				R CAGTCCGCCTAGAAGCATTT
Cancer susceptibility candidate 3	*Casc3*	Endogenous Control	NM_001098069.1	F GGACCTCCACCTCAGTTCAA
				R GTCTTTGCCGTTGTGATGAA
Glyceraldehyde-3-phosphate dehydrogenase	*GAPDH*	Endogenous Control	NM_001034034.1	F CGACTTCAACAGCGACACTC
				R TTGTCGTACACAAGGAAATGAGC

**Table 3:** Influence of protein supplementation during mid-gestation on plasma hormones and metabolites in beef cows.

	Maternal Nutrition		Calf Sex		SEM	*p*-Value		
	CON	SUP	Female	Male		MN	CS	MN × CS
	(*n* = 26)	(*n* = 26)	(*n* = 22)	(*n* = 30)				
Measurements at 200 days of gestation (end of supplementation period)								
IGF-1, Ng/mL	61.9	66.9	62.6	66.2	2.00	0.06	0.18	0.64
Insulin, µIU/mL	6.70	10.4	8.77	8.37	0.45	<0.01	0.51	0.43
Glucose, mg/dL	46.9	54.7	51.1	50.5	1.31	<0.01	0.71	0.62
Urea, mg/dL	36.1	36.4	36.2	36.2	2.16	0.85	0.94	0.78
NEFA, mmol/L	0.11	0.10	0.11	0.10	0.01	0.56	0.50	0.63
BHBA, mmol/L	0.34	0.26	0.29	0.31	0.04	0.08	0.59	0.93
Measurements at 270 days of gestation (pre-partum)								
IGF-1, Ng/mL	58.8	62.09	59.6	61.3	3.20	0.20	0.48	0.33
Insulin, µIU/mL	6.58	9.05	7.78	7.85	0.59	<0.01	0.87	0.14
Glucose, mg/dL	48.4	53.3	52.5	49.1	1.90	0.05	0.18	0.79
Urea, mg/dL	35.4	30.6	33.4	32.6	3.15	0.09	0.70	0.61
NEFA, mmol/L	0.10	0.08	0.09	0.10	0.90	0.01	0.42	0.07
BHBA, mmol/L	0.40	0.48	0.45	0.42	0.05	0.10	0.57	0.12

**Table 4:** Influence of protein supplementation during mid-gestation on the plasma concentration of total amino acids, and glycogenic, ketogenic, and glycetogenic amino acids in beef cows, expressed in nmol/mL.

	Maternal Nutrition		Calf Sex		SEM	*p*-Value		
	CON	SUP	Female	Male		MN	CS	MN × CS
	(*n* = 26)	(*n* = 26)	(*n* = 22)	(*n* = 30)				
Measurements at 200 days of gestation (end of supplementation period)								
Total AA	1019	1533	1107	1445	128	0.03	0.34	0.10
Essential AA	611	662	647	626	66.1	0.11	0.66	0.31
Non-essential AA	579	834	621	791	66.7	0.02	0.16	<0.01
Glucogenic AA	788	966	842	911	71.9	0.10	0.44	0.08
Ketogenic AA	141	132	152	120	17.6	0.69	0.15	0.13
Glucogenic and ketogenic AA	400	450	426	424	37.9	0.49	0.48	0.65
Branched-chain AA	355	351	383	323	47.1	0.95	0.30	0.26
Measurements at 270 days of gestation (pre-partum)								
Total AA	1121	1699	1226	1583	129	0.01	0.47	0.17
Essential AA	689	802	761	730	53.5	0.07	0.50	0.81
Non-essential AA	678	887	711	853	71.2	0.09	0.21	0.19
Glucogenic AA	981	1004	1027	957	62.7	0.76	0.36	0.69
Ketogenic AA	202	156	212	136	54.8	0.17	0.03	0.15
Glucogenic and ketogenic AA	417	529	455	490	31.1	<0.01	0.35	0.51
Branched-chain AA	619	372	661	330	86.3	0.36	0.05	0.06

**Table 5:** Influence of protein supplementation during mid-gestation on the plasma concentration of amino acids in beef cows, expressed in nmol/mL.

Item	Maternal Nutrition		Calf Sex		SEM	*p*-Value		
	CON	SUP	Female	Male		MN	CS	MN × CS
	(*n* = 26)	(*n* = 26)	(*n* = 22)	(*n* = 30)				
Measurements at 200 days of gestation (end of supplementation period)								
Essential AA								
Arginine	70.8	93.7	79.8	84.7	13.0	0.16	0.75	0.59
Phenylalanine	78.0	74.4	88.5	63.9	11.7	0.80	0.10	0.08
Histidine	97.4	89.1	102	84.3	11.5	0.55	0.22	0.68
Isoleucine	125	122	138	108.3	18.3	0.90	0.19	0.31
Methionine	28.0	28.4	30.4	25.9	4.06	0.94	0.37	0.47
Leucine	122	117	129	110	12.2	0.76	0.21	0.19
Tryptophan	25.6	39.3	32.6	31.3	4.47	0.02	0.95	0.94
Non-essential AA								
Aspartate	15.5	21.5	18.8	18.2	4.39	0.26	0.90	0.73
Glutamate	142	122	132	131	19.4	0.41	0.98	0.81
Asparagine	6.87	8.24	8.16	6.95	1.23	0.39	0.42	0.90
Serine	82.2	88.0	76.1	94.2	12.4	0.71	0.24	0.10
Glutamine	177	257	191.3	233	45.0	0.11	0.45	0.09
Tyrosine	43.4	59.9	48.3	55.0	4.37	<0.01	0.21	0.25
Alanine	229	282	257	255	19.3	0.03	0.94	0.15
Measurements at 270 days of gestation (pre-partum)								
Essential AA								
Arginine	65.3	85.3	69.5	81.1	10.4	0.12	0.36	0.23
Phenylalanine	47.7	63.2	57.3	53.7	2.88	0.03	0.58	0.84
Histidine	94.9	101	96.7	99.5	9.66	0.59	0.81	0.48
Isoleucine	191	121	196	116	22.5	0.69	0.20	0.03
Methionine	44.2	27.9	47.5	26.6	5.18	0.36	0.02	0.05
Leucine	122	156.2	142.6	136	5.97	<0.01	0.52	0.67
Tryptophan	21.6	31.3	27.7	25.3	3.52	0.02	0.55	0.69
Non-essential AA								
Aspartate	9.22	9.31	8.54	8.90	4.62	0.89	0.64	0.96
Glutamate	286	317	299	304	26.0	0.33	0.87	0.12
Asparagine	25.9	27.3	25.4	26.5	17.4	0.70	0.71	0.72
Serine	107	104	100	108	13.8	0.84	0.51	0.24
Glutamine	98.3	87.7	87.9	98.2	12.2	0.47	0.49	0.89
Tyrosine	37.8	55.9	42.5	51.2	2.85	<0.01	0.02	0.26
Alanine	211	287	244	253	25.2	0.02	0.77	0.97

## Data Availability

Not applicable.
